# Co-Circulation of Canine Coronavirus I and IIa/b with High Prevalence and Genetic Diversity in Heilongjiang Province, Northeast China

**DOI:** 10.1371/journal.pone.0146975

**Published:** 2016-01-15

**Authors:** Xinyu Wang, Chunqiu Li, Donghua Guo, Xinyu Wang, Shan Wei, Yufei Geng, Enyu Wang, Zhihui Wang, Xiwen Zhao, Mingjun Su, Qiujin Liu, Siyao Zhang, Li Feng, Dongbo Sun

**Affiliations:** 1 College of Animal Science and Veterinary Medicine, Heilongjiang Bayi Agricultural University, No. 5 Xinfeng Road, Sartu District, Daqing 163319, P.R. China; 2 National Key Laboratory of Veterinary Biotechnology, Harbin Veterinary Research Institute of the Chinese Academy of Agricultural Sciences, No. 427 Maduan Street, Nangang District, Harbin 150001, P.R. China; Faculty of Biochemistry Biophysics and Biotechnology, Jagiellonian University, POLAND

## Abstract

To trace the evolution of canine coronavirus (CCoV), 201 stool samples from diarrheic dogs in northeast China were subjected to reverse transcription-polymerase chain reactions (RT-PCRs) targeting the partial M and S genes of CCoV, followed by an epidemiological analysis. M gene RT-PCRs showed that 28.36% (57/201) of the samples were positive for CCoV; of the 57 positive samples, CCoV-I and CCoV-II accounted for 15.79% (9/57) and 84.21% (48/57), respectively. A sequence comparison of the partial M gene revealed nucleotide homologies of 88.4%–100% among the 57 CCoV strains, and 88.7%–96.2% identity between the 57 CCoV strains and the Chinese reference strain HF3. The CCoV-I and CCoV-II strains exhibited genetic diversity when compared with reference strains from China and other countries. The 57 CCoV strains exhibited high co-infection rates with canine kobuvirus (CaKV) (33.33%) and canine parvovirus-2 (CPV-2) (31.58%). The CCoV prevalence in diarrheic dogs differed significantly with immunization status, regions, seasons, and ages. Moreover, 28 S genes were amplified from the 57 CCoV-positive samples, including 26 CCoV-IIa strains, one CCoV-IIb strain, and one CCoV-I strain. A sequence comparison of the partial S gene revealed 86.3%–100% nucleotide identity among the 26 CCoV-IIa strains, and 89.6%–92.2% identity between the 26 CCoV-IIa strains and the Chinese reference strain V1. The 26 CCoV-IIa strains showed genetic diversity when compared with reference strains from China and other countries. Our data provide evidence that CCoV-I, CCoV-IIa, and CCoV-IIb strains co-circulate in the diarrhoetic dogs in northeast China, high co-infection rates with CaKV and CPV-2 were observed, and the CCoV-II strains exhibited high prevalence and genetic diversity.

## Introduction

Canine coronavirus (CCoV) was first recognized as an enteric pathogen of dogs in 1971 [1]. CCoV is a common infection in young dogs, particularly those housed in large groups [2–5]. CCoV is an enveloped, single-stranded, positive-sense RNA virus, and it belongs to the family *Coronaviridae*, subfamily *Coronavirinae*, genus *Alphacoronavirus*, species *Alphacoronavirus-1* [6]. CCoV consists of two distinct genotypes, CCoV-I and CCoV-II; the CCoV-II viruses are further divided into two subtypes CCoV-IIa and CCoV-IIb [3, 7]. The S protein of CCoV is a glycoprotein peplomer on the viral surface, and it plays an important role in the induction of neutralizing antibodies, specific receptor binding, and cell membrane fusion. Accumulating reports attributed that the increase in the severity of CCoV infections in dogs and the emergence of CCoV variants to potential recombination events within the S gene, which occur when a host is co-infected with different CCoV types [8–10]. Therefore, CCoV has received much attention as an emerging cause of infectious disease in dogs [4, 11–16].

CCoV infection is a leading causes of diarrhea in dog population in China. Wang et al. (2006) reported that CCoV-II infections were very common in domestic dog, fox, and raccoon-dog populations in China [17]. Ma et al. (2008) reported the molecular characterization of the 9.36-kb 3′ region of the CCoV 1–71 strain [18]. Gao et al. (2009) reported the isolation and identification of a CCoV strain from giant pandas in China [19]. However, information about the epidemiology of CCoVs in China is not available in the past five years. In the current study, we conducted a molecular epidemiologic investigation of CCoV in Heilongjiang province, northeast China. Moreover, the genetic evolution and co-infection of the identified CCoV strains were analyzed. Our aim was to provide insights into the epidemiology and genetic diversity of the CCoV strains circulating in northeast China.

## Material and Methods

### Ethics Statement

The animal experiment, sampling, was approved by the Animal Care and Use Committee of the Harbin Veterinary Research Institute, Chinese Academy of Agricultural Sciences, China. The sampling and data publication also were approved by animal’s owners. The field study did not involve endangered or protected species. No specific permissions were required for locations of samples because the samples were collected from public areas or non-protection areas.

### Sampling

A total of 201 fecal samples were collected in the form of rectal swabs of dogs with diarrhea from animal hospitals in the Harbin, Daqing, and Mudanjiang districts of Heilongjiang province in northeast China from May 2014 to April 2015, using 3.5-ml commercial virus sampling tubes (YOCON Biological Technology Co. Ltd., Beijing, China). For all samples, animal age, animal breed, animal gender, collection date, and vaccination were recorded, respectively. Of the 201 samples, 141 were collected in Harbin, 20 were collected in Daqing, and 40 were collected in Mudanjiang. All rectal swab samples were stored at −80°C, and they were also used for etiological investigations in our other studies [20, 21].

### RNA extraction

After 1 mL of fecal samples was centrifuged at 1,500 × *g* for 10 min at 4°C, the supernatant of each sample was transferred to a 1.5-ml Eppendorf tube. Viral RNA was extracted from each sample using the TIANamp Virus RNA Kit (Tiangen Biotech Co., Ltd., Beijing, China) according to the manufacturer’s instructions. The extracted RNA were stored at –80°C.

### Detection and sequence analysis of CCoV

Molecular detection of CCoV was conducted using reverse transcription-polymerase chain reaction (RT-PCR) targeting a 409-bp fragment of the M gene of CCoV that was described by Pratelli et al. (1999) [22]. The S gene fragments used for CCoV genotyping/subtyping, including a 346-bp fragment of CCoV-I, a 694-bp fragment of CCoV-IIa, and a 370-bp fragment of CCoV-IIb, were amplified using the primers EL1F/EL1R, S5F/S6R, and CEPol-1/TGSP-2, respectively [23, 24]. Briefly, first-strand cDNA was synthesized using random primers (six nucleotides) using Moloney murine leukemia virus (RNase H-) reverse transcriptase (Novoprotein Scientific Inc., Shanghai, China) according to the manufacturer’s instructions. In this study, the EmeraldAmp PCR Master Mix (2× Premix) (TaKaRa Biotechnology Co., Ltd., Dalian, China), and the Applied Biosystems GeneAmp PCR System 9700 thermal cycler (Thermo Fisher Scientific, Waltham, MA, USA) were used for PCR amplifications of all target genes. Other PCR amplification conditions were performed according to the protocols described by Pratelli et al. (2004) [23] and Erles and Brownlie (2009) [24]. The purified PCR products were directly subjected to Sanger sequencing, and each sample was sequenced three times. Sequence analysis was performed using the EditSeq program in the Lasergene DNASTAR^™^ version 5.06 software (DNASTAR Inc., Madison, WI, USA). All nucleotide sequences generated in our study were submitted to GenBank under accession numbers KT192642–KT192698 for the M gene of the 57 CCoV strains, KT222969–KT222994 for the S gene of the 26 CCoV-IIa strains, KT222995 for the S gene of the CCoV-I strain, and KT222996 for the S gene of the CCoV-IIb strain.

### Phylogenetic analysis

For the phylogenetic analysis, partial sequences of the M and S genes of CCoV strains, including CCoV-I, CCoV-IIa, and CCoV-IIb strains, were retrieved from GenBank. To construct the phylogenetic trees, a multiple alignment of all target sequences was performed using ClustalX program version 1.83. Furthermore, phylogenetic trees of all target sequences were generated from the ClustalX-generated alignments by MEGA6.06 software using the neighbor-joining method [25]. Neighbor-joining phylogenetic trees were built with the p-distance model, 1000 bootstrap replicates, and, otherwise, the default parameters in MEGA 6.

### Screening for canine enteric pathogens

The 57 CCoV-positive samples were screened for CPV-2, CaKV, canine astrovirus (CaAstV), canine norovirus (CNoV), canine bocavirus (CBoV), and Group A-Rotavirus (CRV-A) by either PCR or RT-PCR, followed by sequencing, according to previously described protocols [26–30]. All nucleotide sequences generated in our study were submitted to GenBank, and the accession numbers of the target genes of co-infected viruses are shown in S1 Table.

## Results

### Investigation and genotyping of CCoV

The characteristics of the 57 CCoV-positive dogs, the genotyping of 57 CCoV strains, and the amino acid substitutions of M protein of 57 CCoV strains are shown in S1 Table, and a further analysis of the 57 CCoV-positive samples is shown in Table 1. Nucleotide sequences of the partial M gene of the 57 CCoV strains identified in our study were shown in Supporting Information (S1 Fig). Of the 201 samples, 57 samples (28.36%) were positive for CCoV following RT-PCR amplification of the partial M gene, and the CCoV-positive rates of the Harbin, Daqing, and Mudanjiang districts were 26.95%, 50%, and 22.5%, respectively (Table 1). Of the 57 CCoV-positive samples, CCoV-I and CCoV-II accounted for 15.79% (9/57) and 84.21% (48/57), respectively; immunized animals and non-immunized animals accounted for 43.86% and 36.84%, respectively; 56.14% (32/57) were collected from October to December, and 66.66% (38/57) were collected from dogs aged from 2 to 4 months; the total co-infection rate of CCoV with any viruses reached 56.14%, and CaKV and CPV-2 accounted for 33.33% (19/57) and 31.58% (18/57), respectively (Table 1). Of the 57 CCoV strains, only 28 S gene sequences were successfully amplified, of which 26 belonged to CCoV-IIa, one to CCoV-IIb, and one to CCoV-I (S1 Table). Nucleotide sequences of the partial S gene of CCoV strains identified in our study were shown in Supporting Information (S2 Fig).

**Table 1:** Further analysis of the CCoV-positive samples.

	Harbin	Daqing	Mudanjiang	Total
**Numbers of sample**	141	20	40	201
**Positive rate for CCoV**	26.95% (38/141)	50% (10/20)	22.5% (9/40)	28.36% (57/201)
**Genotyping of CCoV based on the partial M gene**				
I	13.16% (5/38)	10% (1/10)	33.33% (3/9)	15.79% (9/57)
II	86.84% (33/38)	90% (9/10)	66.67% (6/9)	84.21% (48/57)
**Vaccination**^#^				
Yes	52.63% (20/38)	20% (2/10)	33.33% (3/9)	43.86% (25/57)
No	34.21% (13/38)	50% (5/10)	33.33% (3/9)	36.84% (21/57)
**Collection data**				
Jan. to Mar.	5.26% (2/38)	60% (6/10)	22.22% (2/9)	17.54% (10/57)
Apr. to Jun.	2.63% (1/38)	—	11.11% (1/9)	3.51% (2/57)
Jul. to Sep.	31.58% (12/38)	10% (1/10)	—	22.81% (13/57)
Oct. to Dec.	60.53% (23/38)	30% (3/10)	66.67% (6/9)	56.14% (32/57)
**Age**				
0< Age ≤1M	—	10% (1/10)	—	1.75% (1/57)
1M< Age ≤2M	13.16% (5/38)	50% (5/10)	33.33% (3/9)	22.81% (13/57)
2M< Age ≤3M	42.11% (16/38)	30% (3/10)	22.22% (2/9)	36.84% (21/57)
3M< Age ≤4M	34.21% (13/38)	—	44.44% (4/9)	29.82% (17/57)
**Coinfection of CCoV with other enteric viruses**				
Single infection				
CPV-2	36.84% (14/38)	20% (2/10)	22.22% (2/9)	31.58% (18/57)
CaKV	39.47% (15/38)	30% (3/10)	11.11% (1/9)	33.33% (19/57)
CBoV	2.63% (1/38)	—	22.22% (2/9)	5.26% (3/57)
Mixed infection				
CPV-2+CaKV	21.05% (8/38)	—	—	14.04% (8/57)
CPV-2+CBoV+CaKV	2.63% (1/38)	—	—	1.75% (1/57)
Total co-infection				
CaKV, CPV-2, CBoV, CPV-2+CaKV or CPV-2+CBoV+CaKV	57.89% (22/38)	50% (5/10)	55.56% (5/9)	56.14% (32/57)
**Identity of partial M gene of CCoV identified in our study**				
Nuleotides				
I	95.7%-99.1%	—	97.4%-100%	94.8%-100%
II	97.4%-100%	97.4%-100%	97.7%-100%	96.8%-100%
I+II	88.4%-100%	92.5%-100%	89.6%-100%	88.4%-100%
Amino acids				
I	97.4%-100%	—	100%	94.8%-100%
II	100%	98.3%-100%	100%	98.3%-100%
I+II	94.8%-100%	93.9%-100%	95.7%-100%	93%-100%
**Compared with M gene of the HF3 strain from China (AY864661)**				
Nuleotides				
I+II	88.7%-96.2%	91.3%-95.9%	89%-95.9%	88.7%-96.2%
Amino acids				
I+II	92.2%-97.4%	93%-97.4%	93%-97.4%	92.2%-97.4%
**Identity of partial S gene of CCoV identified in our study**				
Nuleotides				
IIa	89.3%-100%	87.7%-100%	—	86.3%-100%
Amino acids				
IIa	92.1%-100%	87.6%-100%	—	87.6%-100%
**Compared with S gene of the V1 strain from China (AY390342)**				
Nuleotides				
IIa	89.6%-92.2%	89.9%-92.2%	—	89.6%-92.2%
Amino acids				
IIa	95.5%-97.5%	91.1%-97.5%	—	91.1%-97.5%
**Amino acid substitution of partial M protein of CCoV identified in our study**				
^178^Thr/Val				
I (Val→Thr)	100% (5/5)	0 (0/1)	100% (3/3)	88.89% (8/9)
II (Thr→Val)	100% (33/33)	100% (9/9)	100% (6/6)	100% (48/48)
^198^Met/Ile				
I (Ile→Met)	100% (5/5)	100% (1/1)	100% (3/3)	100% (9/9)
II (Met→Ile)	100% (33/33)	100% (9/9)	100% (6/6)	100% (48/48)
^206^His/Asn				
I (Asn→His)	100% (5/5)	100% (1/1)	100% (3/3)	100% (9/9)
II (His→Asn)	100% (33/33)	100% (9/9)	100% (6/6)	100% (48/48)
^228^Gln/Lys				
I (Lys→Gln)	100% (5/5)	0 (0/1)	100% (3/3)	88.89% (8/9)
II (Gln→Lys)	100% (33/33)	100% (9/9)	100% (6/6)	100% (48/48)

^#^The animals are vaccinated for CPV-2, canine distemper virus, canine parainfluenza virus, canine adenovirus type 1, and canine adenovirus type 2.

### Sequences and phylogenetic analysis of CCoV

The sequence comparison of the partial M gene revealed nucleotide homologies of 88.4%–100% and amino acids homologies of 93%–100% among the 57 CCoV strains, while nucleotide and amino acid homologies of 88.7%–96.2% and 92.2%–97.4%, respectively, were observed between the 57 CCoV strains and the Chinese reference strain HF3. Of the 57 CCoV strains, the nine CCoV-I strains exhibited 94.8%–100% nucleotide identities and 94.8%–100% amino acid identities, and the 48 CCoV-II strains showed 96.8%–100% nucleotide identities and 98.3%–100% amino acid identities (Table 1). A total of 12 amino acid substitutions were found in the partial M protein (S1 Table); the four substitutions, Thr178Val, Met198Ile, His206Asn, and Gln228Lys, occurred in all identified CCoV-II strains (48/48), and two substitutions, Ile198Met and Asn206His, occurred in all identified CCoV-I strains (9/9) (Table 1). The sequence comparison of the partial S gene revealed nucleotide homologies of 86.3%–100% and amino acids homologies of 87.6%–100% among the the 26 CCoV-IIa strains, and nucleotide and amino acid homologies of 89.6%–92.2% and 91.1%–97.5%, respectively, between the 26 CCoV-IIa strains and the Chinese reference strain V1 (Table 1).

**Fig 1:**
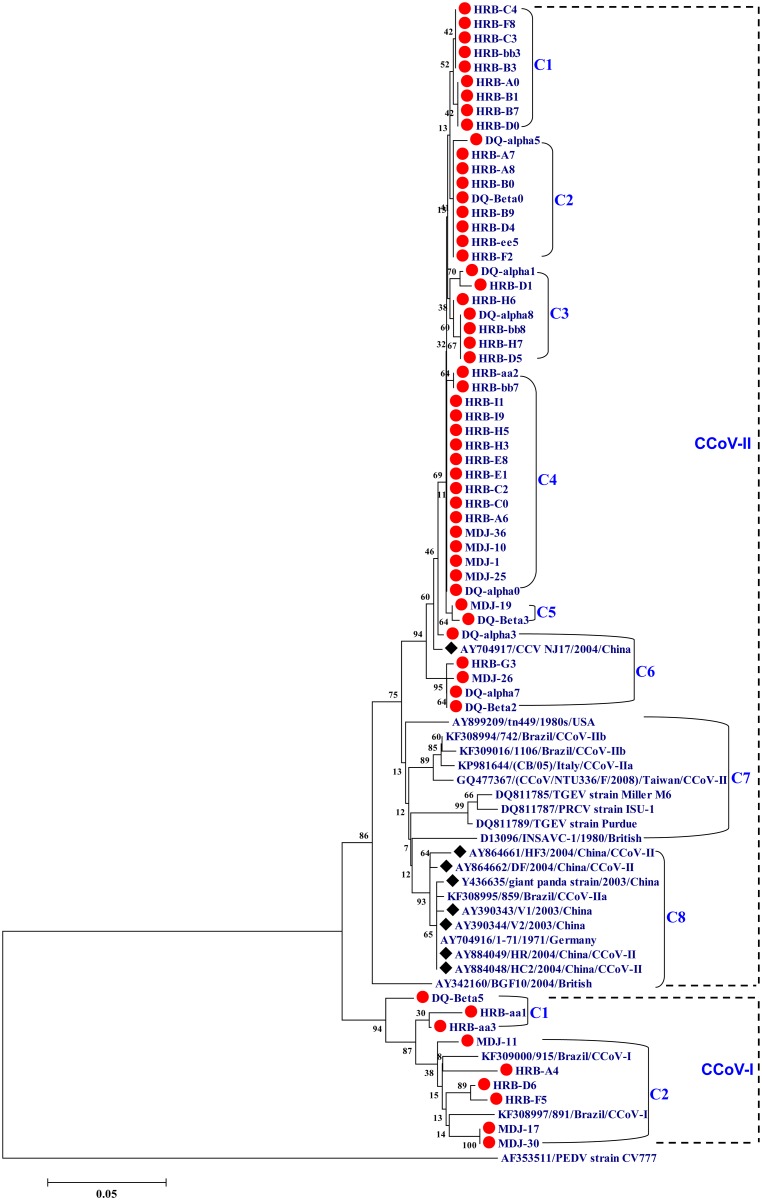
Phylogenetic analysis of CCoV strains using the nucleotide sequences of the partial M gene of CCoV. Red circles represent the CCoV strains identified in our study, and black diamonds represent the Chinese CCoV reference strains.

**Fig 2:**
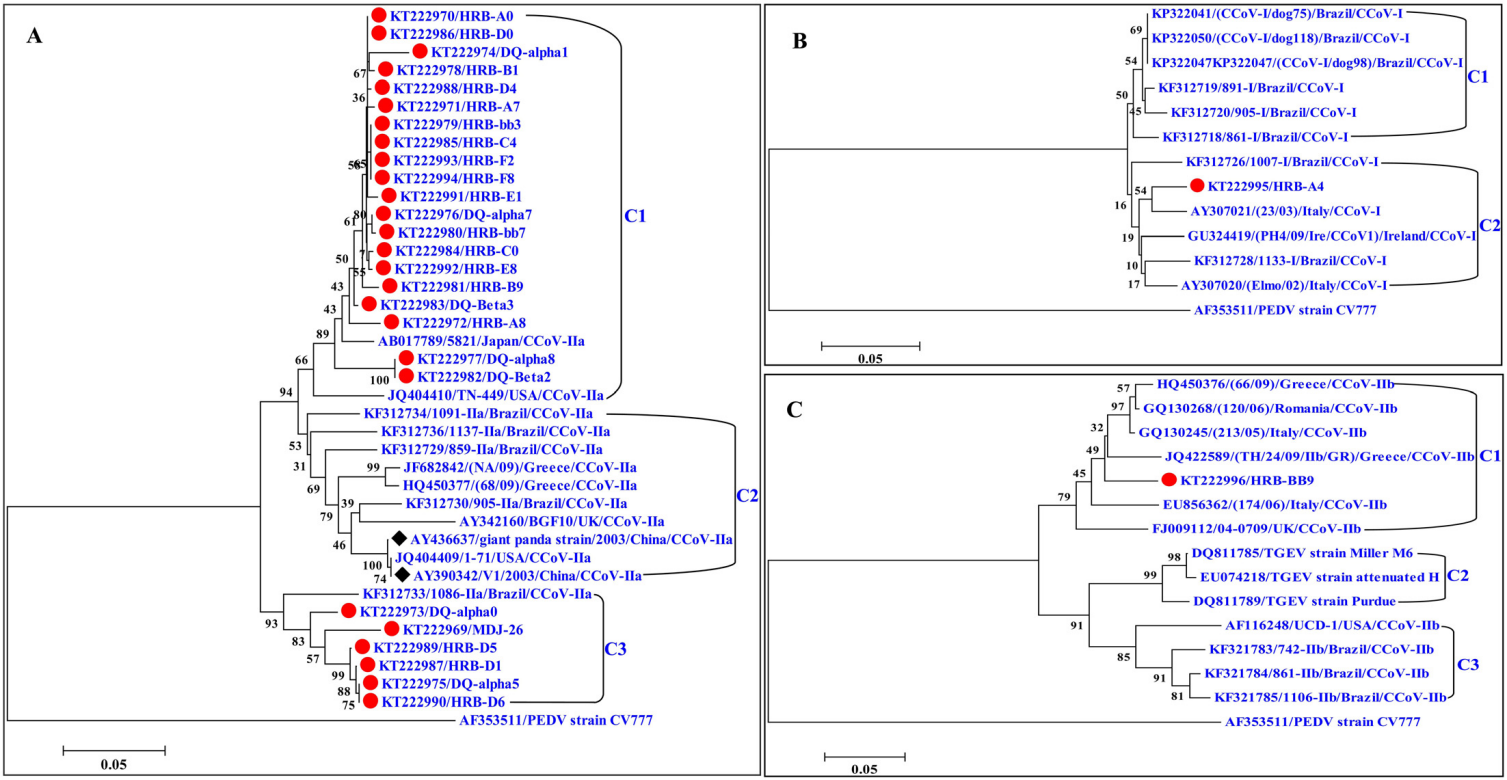
Phylogenetic analysis of CCoV strains on the basis of partial S gene sequences. (A) Phylogenetic tree generated using the nucleotide sequences of the partial S gene of CCoV-IIa. (B) Phylogenetic tree generated using the nucleotide sequences of the partial S gene of CCoV-I. (C) Phylogenetic tree generated using the nucleotide sequences of the partial S gene of CCoV-IIb. Red circles represent the CCoV strains identified in our study.

A phylogenetic analysis using the nucleotide sequences of the M gene revealed that the nine CCoV-I strains formed two clusters (C1 and C2), while the 48 CCoV-II strains formed six clusters (C1–C6). The 48 CCoV-II strains only exhibited a close relationship to one Chinese reference strain, NJ17, and differed genetically from most of the reference strains from China and other countries (Fig 1). A phylogenetic analysis using partial S gene sequences demonstrated that the 26 CCoV-IIa strains were closely related to three reference strains, 5281 (Japan), TN-449 (USA), and 1086-IIa (Brazil), and differed genetically from reference strains from China and other countries (Fig 2A). The CCoV-I strain HRB-A4 showed a close relationship to the reference strain from Italy (Fig 2B), while the CCoV-IIb strain HRB-BB9 was closely related to reference strains from European countries (Fig 2C).

## Discussion

In our study, 57 of 201 samples (28.36%) were positive for CCoV, and the CCoV-positive rate differed among the three districts of Heilongjiang province (26.95% for Harbin, 50% for Daqing, and 22.5% for Mudanjiang). The CCoV-positive rate in feces has been reported to be 43.75% (21/48), 32.83% (22/67), 50.46% (55/109), 54.39% (31/57), 12% (30/250), 51.3% (20/39), 80% (8/10), 60% (3/5), 38.09% (8/21), 12.5% (1/8), 36.17% (17/47), and 11.54% (3/26) in China [17], the Republic of Korea [31], Japan [32], Albania [33], Brazil [11], Italy, Belgium, the Netherlands, Germany, the United Kingdom, Spain, and France, respectively [34]. These data demonstrated that CCoV infections showed clear differences among the geographical regions. The total CCoV-positive rate reported here was lower than that in a previous report in 2006 in China [17]. In our study, CCoV-II, accounting for 84.21% of the 57 CCoV strains, was the predominant CCoV type in northeast China, which is line with most reports [11, 17, 34].

Mixed infections of canine enteric viruses frequently occur in diarrheic dogs. In our study, the total co-infection rate of CCoV with one or more CaKV, CPV-2, and CBoV strains was 56.14%; single co-infections with CaKV, CPV-2, and CBoV accounted for 33.33%, 31.58%, and 5.26%, respectively. Co-infections between CCoV and CPV-2 have been documented in dog populations in Western Europe, Japan, and Albania [32–34]. However, the high co-infection rate of CCoV with CaKV has not been reported in China and other countries. CaKV had been reported to be associated with severe enteritis in a litter of puppies [13]. It is speculated that the high prevalence of co-infections of CCoV with CaKV and CPV-2 may be associated with the occurrence of viral diarrhea in dogs in northeast China. Further studies should be conducted to understand the effect of the high co-infection rate with CaKV on the severity of clinical symptoms of CCoV infections.

In our study, CCoV-positive rate showed clear differences among seasons and ages. The high prevalence of CCoV in the feces of diarrheic dogs aged 2–4 months was partially validated in other studies [11, 32, 33]. The high positive rate (56.14%) of CCoV was found between October and December, which may be associated with seasonal variations in northeast China. At present, most dogs in northeast China are vaccinated for CPV-2, canine distemper virus, canine parainfluenza virus, canine adenovirus type 1, and canine adenovirus type 2. In our study, of the 57 CCoV-positive samples, the immunized and non-immunized animals accounted for 43.86% and 36.84%, respectively. This result demonstrated that vaccination for other canine viruses did not effect CCoV infections in the dog population in Heilongjiang province, northeast China. Given the high prevalence and co-infection rates of CCoV, although it is controversial whether CCoV vaccines provide adequate immunity [35, 36], the immunization for CCoV is recommended in the dog population in northeast China in the future.

Fragments of the M and S genes have been used to genotype CCoV [4, 11, 24, 37]. In our study, the CCoV-I and CCoV-II genotypes of the 57 CCoV strains were successfully identified using fragments of the M gene to construct a phylogenetic tree, coupled with an analysis of amino acids substitutions of the partial M protein. In the phylogenetic tree of the M gene, the 48 CCoV-II strains identified in our study, which formed six clusters (C1–C6), differed genetically from other country’s reference strains and the early Chinese strains (except strain NJ17), and they possessed characteristics of the CCoV strains in northeast China. Meanwhile, the nine CCoV-I strains identified in our study formed two clusters (C1 and C2). These data demonstrated that the CCoV-I and CCoV-II strains identified in our study exhibited genetic diversity. Additionally, of the nine CCoV-I strains, only one co-infection between CCoV-I and CCoV-II was found, accounting for 11.11% (1/9). This result was lower than that in a recent report in Brazil [11]. In the M protein, the Thr178Val, Met198Ile, His206Asn, and Gln228Lys substitutions were common in all identified CCoV-II strains when compared with amino acids substitutions in the partial M protein from all identified CCoV-I strains and reference strains.

Co-infection of CCoV-I and CCoV-II strains has been documented in previous studies [17, 32, 38–41]. To investigate single or multiple infections of CCoV types I, IIa, and IIb, we attempted to amplify three fragments of the S gene of 57 CCoV-positive samples by RT-PCR. Only the S genes of 28 CCoV strains were obtained, of which 26 strains belonged to CCoV-IIa, while the remaining two strains belonged to CCoV-I and CCoV-IIb, respectively. Compared with the reference strains, the partial S genes of the 26 Chinese CCoV-IIa strains exhibit genetic diversity, which is in agreement with the phylogenetic analysis of the M genes identified in our study. The CCoV-IIb strain HRB-BB9 was closely related to CCoV-IIb reference strains from Europe. The CCoV-I strain HRB-A4 clustered with reference strains from Europe, North America and South America. Additionally, it was rarely reported that one co-infection between CCoV-IIa and CCoV-IIb was found in the 28 CCoV-positive samples. The limited data demonstrated that CCoV-I, CCoV-IIa, and CCoV-IIb co-circulated in northeast China, and that CCoV-IIa strains predominated in the dog population.

## Conclusions

The current study is the first to reveal that CCoV-I, CCoV-IIa, and CCoV-IIb strains co-circulate in northeast China, and that there are high co-infection rate with CaKV and CPV-2. CCoV-II strains are predominant, and they exhibit genetic diversity. Resulting data increase our understanding of the evolution of CCoV strains circulating in northeast China, and they provide valuable epidemiological information for further studies of CCoV. However, further studies are necessary to clarify the effect of co-infections and amino acid substitutions on the severity of the clinical symptoms of CCoV infections.

## Supporting Information

S1 Fig Nucleotide sequences of the partial M gene of CCoV strains identified in our study. (TXT)

S2 Fig Nucleotide sequences of the partial S gene of CCoV strains identified in our study. (TXT)

S1 Table Characteristics of the CCoV positive dogs, genotyping of CCoV strains, and amino acid substitution of the partial M protein in northeast China. (DOC)
