# Supplementary material for: Co-Circulation of Canine Coronavirus I and IIa/b with High Prevalence and Genetic Diversity in Heilongjiang Province, Northeast China
Source: PLoS One. 2016 Jan 15;11(1):e0146975. doi: 10.1371/journal.pone.0146975 (PMC4714894; doi:10.1371/journal.pone.0146975)
Supplement: S1 Table — (DOC) [file pone.0146975.s003.doc]

**Table 1** Characteristics of the CCoV positive dogs, genotyping of CCoV strains, and amino acid substitution of the partial M protein in northeast China.

|  | **Strain name** | **Accession No.** | **Collection date** | **Location** | **Age** | **Vaccine** | **Other enteric viruses** | **CCoV genotype** | | **Substitution of amino acid residues in partial M gene of CCoV** | | | | | | | | | | | |
| --- | --- | --- | --- | --- | --- | --- | --- | --- | --- | --- | --- | --- | --- | --- | --- | --- | --- | --- | --- | --- | --- |
|  |  |  |  |  |  |  |  | M gene | S gene | 133  Leu | 139  Val | 167  Leu | 178  Val | 184  Leu | 187  Leu | 198  Ile | 205  Asp | 206  Asn | 218  Arg | 224  Leu | 228  Lys |
| **Reference strains** | 891 | KF308997 | 2008 | Brazil | — | — | — | I | I | — | — | — | Thr | — | — | Met | Glu | His | — | — | Gln |
|  | 915 | KF309000 | 2008 | Brazil | — | — | — | I | I | — | — | — | Thr | — | — | Met | Glu | His | — | — | Gln |
|  | CCoV/NTU336/F/2008 | GQ477367 | 2008 | Taiwan | — | — | — | II | — | — | — | — | — | — | — | — | — | — | — | — | — |
|  | CB/05 | KP981644 | 2005 | Italy | — | — | — | II | IIa | — | — | — | — | — | — | — | — | — | — | — | — |
|  | 859 | KF308995 | 2007 | Brazil | — | — | — | II | IIa | — | Ile | — | — | — | — | — | — | — | — | — | — |
|  | 742 | KF308994 | 2006 | Brazil | — | — | — | II | IIb | — | — | — | — | — | — | — | — | — | — | — | — |
|  | 1106 | KF309016 | 2012 | Brazil | — | — | — | II | IIb | — | — | — | — | — | — | — | — | — | — | — | — |
|  | DF | AY864662 | 2004 | China | — | — | — | II | — | — | Ile | — | — | — | — | — | — | — | — | — | — |
|  | HC2 | AY884048 | 2004 | China | — | — | — | II | — | — | Ile | — | — | — | — | — | — | — | — | — | — |
| **Strains in this study** | DQ-Beta5 | KT192676 | Feb-2015 | DQ | 2M | NA | — | I | NA | — | — | Glu | Ala | — | Phe | Met | — | His | — | — | — |
|  | HRB-aa3 | KT192658 | Sep-2014 | HRB | NA | No | CaKV (KT210395) | I | NA | — | — | Glu | Thr | — | — | Met | — | His | — | — | Gln |
|  | MDJ-11 | KT192644 | Oct-2014 | MDJ | 4M | No | CPV-2c (KT074350) | I | NA | — | — | — | Thr | — | — | Met | Glu | His | — | — | Gln |
|  | MDJ-17 | KT192645 | Dec-2014 | MDJ | 1.5M | NA | CBoV (KR998483) | I | NA | — | — | — | Thr | — | — | Met | Glu | His | — | — | Gln |
|  | MDJ-30 | KT192649 | Jan-2015 | MDJ | 4M | NA | — | I | NA | — | — | — | Thr | — | — | Met | Glu | His | — | — | Gln |
|  | HRB-aa1 | KT192656 | Sep-2014 | HRB | 3.5M | Yes | CPV-2a (KT074276), CaKV (KT210394), CBoV (KR998488) | I | NA | — | — | — | Thr | — | — | Met | Glu | His | — | — | Gln |
|  | HRB-F5 | KT192690 | Nov-2014 | HRB | 4M | Yes | CPV-2b (KT074328) | I | NA | — | — | — | Thr | — | — | Met | Glu | His | — | — | Gln |
|  | HRB-D6 | KT192685 | Oct-2014 | HRB | NA | Yes | CaKV (KT210413) | I | IIa | — | — | — | Thr | Phe | — | Met | Glu | His | — | — | Gln |
|  | HRB-A4 | KT192652 | Sep-2014 | HRB | 3M | NA | CPV-2b (KT074319), CaKV (KT210396) | I | I | Pro | — | — | Thr | — | — | Met | Glu | His | — | — | Gln |
|  | DQ-alpha5 | KT192662 | Feb-2015 | DQ | 3M | NA | CaKV (KT210400) | II | IIa | — | — | — | — | — | — | — | — | — | Ser | Ile | — |
|  | MDJ-1 | KT192642 | Dec-2014 | MDJ | 2M | Yes | CPV-2a (KT074258) | II | NA | — | — | — | — | — | — | — | — | — | — | — | — |
|  | MDJ-10 | KT192643 | Nov-2014 | MDJ | 4M | No | CaKV (KT210390) | II | NA | — | — | — | — | — | — | — | — | — | — | — | — |
|  | MDJ-19 | KT192646 | Nov-2014 | MDJ | 3M | Yes | — | II | NA | — | — | — | — | — | — | — | — | — | — | — | — |
|  | MDJ-25 | KT192647 | Dec-2014 | MDJ | 2M | No | — | II | NA | — | — | — | — | — | — | — | — | — | — | — | — |
|  | MDJ-26 | KT192648 | Apr-2015 | MDJ | 4M | Yes | CBoV (KR998486) | II | IIa | — | — | — | — | — | — | — | — | — | — | — | — |
|  | MDJ-36 | KT192650 | Jan-2015 | MDJ | 3M | NA | — | II | NA | — | — | — | — | — | — | — | — | — | — | — | — |
|  | HRB-A0 | KT192651 | Sep-2014 | HRB | 3M | No | — | II | IIa | — | — | — | — | — | — | — | — | — | — | — | — |
|  | HRB-A6 | KT192653 | Sep-2014 | HRB | 3M | Yes | CPV-2c (KT074339) | II | NA | — | — | — | — | — | — | — | — | — | — | — | — |
|  | HRB-A7 | KT192654 | Sep-2014 | HRB | 3M | No | CaKV (KT210397) | II | IIa | — | — | — | — | — | — | — | — | — | — | — | — |
|  | HRB-A8 | KT192655 | Sep-2014 | HRB | 1.5M | Yes | CPV-2a (KT074280), CaKV (KT210398) | II | IIa | — | — | — | — | — | — | — | — | — | — | — | — |
|  | HRB-aa2 | KT192657 | Sep-2014 | HRB | 3M | Yes | — | II | NA | — | — | — | — | — | — | — | — | — | — | — | — |
|  | DQ-alpha0 | KT192659 | Mar-2015 | DQ | 2M | No | — | II | IIa | — | — | — | — | — | — | — | — | — | — | — | — |
|  | DQ-alpha1 | KT192660 | Dec-2014 | DQ | 3M | No | CPV-2a (KT074273) | II | IIa | — | — | — | — | — | — | — | — | — | — | — | — |
|  | DQ-alpha3 | KT192661 | Jan-2015 | DQ | 2M | No | CaKV (KT210399) | II | NA | — | — | — | — | — | — | — | — | — | — | — | — |
|  | DQ-alpha7 | KT192663 | Mar-2015 | DQ | 2M | Yes | — | II | IIa | — | — | — | — | — | — | — | — | — | — | — | — |
|  | DQ-alpha8 | KT192664 | Sep-2014 | DQ | NA | Yes | — | II | IIa | — | — | — | — | — | — | — | — | — | — | — | — |
|  | HRB-B0 | KT192665 | Oct-2014 | HRB | 3M | Yes | CPV-2a (KT074279), CaKV (KT210404) | II | NA | — | — | — | — | — | — | — | — | — | — | — | — |
|  | HRB-B1 | KT192666 | Sep-2014 | HRB | NA | Yes | CaKV (KT210403) | II | IIa | — | — | — | — | — | — | — | — | — | — | — | — |
|  | HRB-B3 | KT192667 | Sep-2014 | HRB | 3M | Yes | CaKV (KT210405) | II | NA | — | — | — | — | — | — | — | — | — | — | — | — |
|  | HRB-B7 | KT192668 | Oct-2014 | HRB | 3M | Yes | — | II | NA | — | — | — | — | — | — | — | — | — | — | — | — |
|  | HRB-B9 | KT192669 | Oct-2014 | HRB | 3M | Yes | CaKV (KT210407) | II | IIa/ IIb | — | — | — | — | — | — | — | — | — | — | — | — |
|  | HRB-bb3 | KT192670 | Oct-2014 | HRB | 2M | No | CPV-2c (KT074341), CaKV (KT210409) | II | IIa | — | — | — | — | — | — | — | — | — | — | — | — |
|  | HRB-bb7 | KT192671 | Oct-2014 | HRB | 2M | NA | CPV-2a (KT074281) | II | IIa | — | — | — | — | — | — | — | — | — | — | — | — |
|  | HRB-bb8 | KT192672 | Oct-2014 | HRB | 2M | No | CPV-2a (KT074284), CaKV (KT210406) | II | NA | — | — | — | — | — | — | — | — | — | — | — | — |
|  | DQ-Beta0 | KT192673 | Dec-2014 | DQ | 1.5M | No | CPV-2b (KT074336) | II | NA | — | — | — | — | — | — | — | — | — | — | — | — |
|  | DQ-Beta2 | KT192674 | Feb-2015 | DQ | 1M | NA | — | II | IIa | — | — | — | — | — | — | — | — | — | — | — | — |
|  | DQ-Beta3 | KT192675 | Nov-2014 | DQ | 3M | No | CaKV (KT210402) | II | IIa | — | — | — | — | — | — | — | — | — | — | — | — |
|  | HRB-C0 | KT192677 | Oct-2014 | HRB | 4M | No | — | II | IIa | — | — | — | — | — | — | — | — | — | — | — | — |
|  | HRB-C2 | KT192678 | Oct-2014 | HRB | NA | No | CPV-2a (KT074281), CaKV (KT210410) | II | NA | — | — | — | — | — | — | — | — | — | — | — | — |
|  | HRB-C3 | KT192679 | Oct-2014 | HRB | 3.5M | No | — | II | NA | — | — | — | — | — | — | — | — | — | — | — | — |
|  | HRB-C4 | KT192680 | Oct-2014 | HRB | 3.5M | Yes | — | II | IIa | — | — | — | — | — | — | — | — | — | — | — | — |
|  | HRB-D0 | KT192681 | Nov-2014 | HRB | 3.5M | Yes | — | II | IIa | — | — | — | — | — | — | — | — | — | — | — | — |
|  | HRB-D1 | KT192682 | Oct-2014 | HRB | 4M | Yes | CaKV (KT210411) | II | IIa | — | — | — | — | — | — | — | — | — | — | — | — |
|  | HRB-D4 | KT192683 | Oct-2014 | HRB | 4M | NA | — | II | IIa | — | — | — | — | — | — | — | — | — | — | — | — |
|  | HRB-D5 | KT192684 | Oct-2014 | HRB | 3.5M | No | — | II | IIa | — | — | — | — | — | — | — | — | — | — | — | — |
|  | HRB-E1 | KT192686 | Nov-2014 | HRB | 3.5M | Yes | CPV-2a (KT074298) | II | IIa | — | — | — | — | — | — | — | — | — | — | — | — |
|  | HRB-E8 | KT192687 | Nov-2014 | HRB | 3M | Yes | CPV-2a (KT074300), CaKV (KT210415) | II | IIa | — | — | — | — | — | — | — | — | — | — | — | — |
|  | HRB-ee5 | KT192688 | Apr-2015 | HRB | 3.5M | No | — | II | NA | — | — | — | — | — | — | — | — | — | — | — | — |
|  | HRB-F2 | KT192689 | Nov-2014 | HRB | 2.5M | Yes | — | II | IIa | — | — | — | — | — | — | — | — | — | — | — | — |
|  | HRB-F8 | KT192691 | Nov-2014 | HRB | 3M | No | CPV-2a (KT074303), CaKV (KT210418) | II | IIa | — | — | — | — | — | — | — | — | — | — | — | — |
|  | HRB-G3 | KT192692 | Feb-2015 | HRB | 3M | Yes | — | II | NA | — | — | — | — | — | — | — | — | — | — | — | — |
|  | HRB-H3 | KT192693 | Jan-2015 | HRB | 3M | No | — | II | NA | — | — | — | — | — | — | — | — | — | — | — | — |
|  | HRB-H5 | KT192694 | Nov-2014 | HRB | 3.5M | Yes | — | II | NA | — | — | — | — | — | — | — | — | — |  |  |  |
|  | HRB-H6 | KT192695 | Dec-2014 | HRB | 1.5M | NA | CaKV (KT210424) | II | NA | — | — | — | — | — | — | — | — | — | — | — | — |
|  | HRB-H7 | KT192696 | Nov-2014 | HRB | 3M | No | — | II | NA | — | — | — | — | — | — | — | — | — | — | — | — |
|  | HRB-I1 | KT192697 | Sep-2014 | HRB | 4M | NA | CPV-2a (KT074308) | II | NA | — | — | — | — | — | — | — | — | — | — | — | — |
|  | HRB-I9 | KT192698 | Sep-2014 | HRB | 3M | Yes | — | II | NA | — | — | — | — | — | — | — | — | — | — | — | — |

***Note****.* M=month; for location, MDJ=Mudanjiang, HRB=Harbin, DQ=Daqing.
